# Ultrasound for the Anesthesiologists: Present and Future

**DOI:** 10.1155/2013/683685

**Published:** 2013-11-20

**Authors:** Abdullah S. Terkawi, Dimitrios Karakitsos, Mahmoud Elbarbary, Michael Blaivas, Marcel E. Durieux

**Affiliations:** ^1^Department of Anesthesiology, University of Virginia Health System, Charlottesville, VA 22903, USA; ^2^Department of Anesthesiology, King Fahad Medical City, Riyadh, Saudi Arabia; ^3^Department of Internal Medicine, University of South Carolina, School of Medicine, Columbia, SC, USA; ^4^Department of Cardiac Sciences, Critical Care & National and Gulf Center for Evidence Base Health Practice, King Saud bin Abdulaziz University for Health Sciences, Riyadh, Saudi Arabia; ^5^Department of Emergency Medicine, University of South Carolina, School of Medicine, Columbia, SC, USA

## Abstract

Ultrasound is a safe, portable, relatively inexpensive, and easily accessible imaging modality, making it a useful diagnostic and monitoring tool in medicine. Anesthesiologists encounter a variety of emergent situations and may benefit from the application of such a rapid and accurate diagnostic tool in their routine practice. This paper reviews current and potential applications of ultrasound in anesthesiology in order to encourage anesthesiologists to learn and use this useful tool as an adjunct to physical examination. Ultrasound-guided peripheral nerve blockade and vascular access represent the most popular ultrasound applications in anesthesiology. Ultrasound has recently started to substitute for CT scans and fluoroscopy in many pain treatment procedures. Although the application of airway ultrasound is still limited, it has a promising future. Lung ultrasound is a well-established field in point-of-care medicine, and it could have a great impact if utilized in our ORs, as it may help in rapid and accurate diagnosis in many emergent situations. Optic nerve sheath diameter (ONSD) measurement and transcranial color coded duplex (TCCD) are relatively new neuroimaging modalities, which assess intracranial pressure and cerebral blood flow. Gastric ultrasound can be used for assessment of gastric content and diagnosis of full stomach. Focused transthoracic (TTE) and transesophageal (TEE) echocardiography facilitate the assessment of left and right ventricular function, cardiac valve abnormalities, and volume status as well as guiding cardiac resuscitation. Thus, there are multiple potential areas where ultrasound can play a significant role in guiding otherwise blind and invasive interventions, diagnosing critical conditions, and assessing for possible anatomic variations that may lead to plan modification. We suggest that ultrasound training should be part of any anesthesiology training program curriculum.

## 1. Introduction

Anesthesiologists require quick and accurate diagnostic tools for the effective management of emergencies. Ultrasound (US) is a safe, easily accessible point-of-care imaging modality that is being increasingly adopted in modern anesthesiology practice. As physician-performed ultrasound becomes more practical and practiced, it is important to assure that anesthesiologists are aware of the expanding applications of this technology and the status of its use. Current and potential future applications of US in anesthesiology are summarized as follows:

1. regional anesthesia;
2. neuraxial and chronic pain procedures;
3. vascular access;
4. airway assessment;
5. lung ultrasound;
6. ultrasound neuro-monitoring;
7. gastric ultrasound;
8. focused transthoracic echo (TTE);
9. transesophageal echo (TEE) and Doppler (Beyond the scope of discussion in this paper).

This paper highlights current and potential future applications of ultrasound in the field of anesthesiology. Our objective is to increase awareness of the current benefits and limitations of selected ultrasound applications that may be relevant to anesthesiologists. We aim to encourage practitioners to acquire appropriate training that will allow them to apply well-established ultrasound techniques in their routine practice, to assure that they are aware of constraints and limitations in various settings, and to remain alert for the development of ultrasound techniques that are a focus of ongoing research.

## 2. Principles of Ultrasound

**Figure 1:**
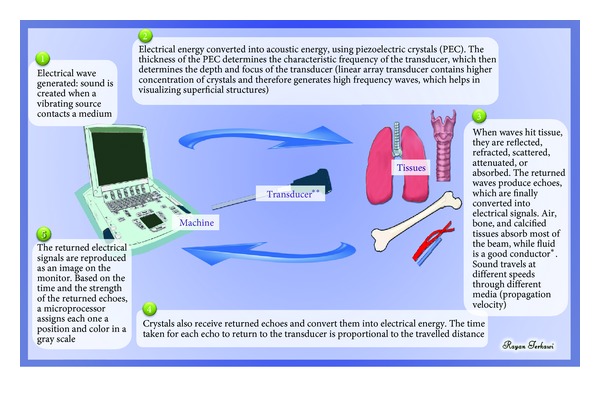
Concept of ultrasonography: *tissues that allow the beam to pass easily (e.g., containing fluids or blood) create only little echo (hypoechoic) and appear black on the screen, while tissues that allows less beam to pass (e.g., fat and bone) create stronger echoes (hyperechoic) and thus appear white on the screen; **linear transducers have a higher frequency (10–15 MHz) and are usually used for superficial structures; curved transducers have a lower frequency (4–8 MHz) and are usually used for deeper structures.

Ultrasound (“extrasound”) refers to the use of sound waves (typically from 2 to 15 MHz, but in modern probes up to 22 MHz), which are above the frequency of those sound waves that can be heard by the human ear (20 to 20,000 Hz range). The concept of ultrasound is explained in Figure 1 [1–3]. 

Several basic and advanced modes of ultrasound imaging exist, but B-mode, M-mode, and color-Doppler are those most commonly used in anesthesiology [3]. B-mode (brightness) is the main mode of any ultrasound machine. Each gray scale tomographic image in B-mode is composed of pixels with brightness depending on the intensity of the echo that is received from the corresponding location in the body. This mode is used to evaluate and scan organs in real time [4, 5]. M-mode (motion) displays the movement of structures along a single line (axis of the ultrasound beam) chosen by the operator. M-mode is used for evaluation of heart wall or valve motion (echocardiography), hemodynamic status (vena cava analysis), and documentation of lung sliding or movement of the diaphragm [5]. 

Doppler modes detect frequency shifts that are created by sound reflections of a moving target (Doppler effect). It uses the change in pitch of the sound waves to provide information about blood flow [6]. Three Doppler techniques are commonly used. (1) Color flow Doppler: this gives an image of the blood vessel that represents the speed and direction of blood flow through the vessel. The colors (usually red and blue) denote flow towards and away from the transducer, regardless of the vessel's nature (artery or vein). Power Doppler is a special type of color Doppler. (2) Pulsed wave Doppler (PWD) transmits short pulses of ultrasound and Doppler signals. It allows measurements of blood velocity from a small region, by converting the Doppler sounds into a graph that gives information about the speed and direction of blood flow through the blood vessel. (3) Continuous wave Doppler (CWD) transmits and receives continuous ultrasound waves. The region from which Doppler signals are obtained is determined by the overlap of the transmitted and received ultrasound beams. It is useful for measuring high velocities but with poor ability to localize a flow signal accurately, since the signal can originate from any point along the ultrasound beam.

## 3. Regional Anesthesia

Ultrasound-guided peripheral nerve blockade is perhaps the most popular ultrasound application used by anesthesiologists. It might be the gold standard for regional anesthesia; it allows anesthesiologists to perform regional anesthesia more accurately, and it expands the ability to block smaller nerves and those in more difficult anatomic locations. 

Ultrasound-guided peripheral nerve blocks offer the following advantages: direct observation of the nerves and surrounding structures (e.g., vessels), thus decreasing complications (e.g., accidental intraneural or intravascular injection), and direct observation of local anesthetic spread. The more accurate deposition leads to faster onset and longer duration of block, improves block quality, and allows dose reduction of local anesthetics [7–9]. It has been shown that when peripheral nerves are adequately imaged by ultrasound, the simultaneous use of the nerve stimulator offers no further advantages [7]. 

In children, ultrasound guidance carries similar advantages as for adults and has become more popular recently. However, there is still a shortage of clinical studies comparing the advantages of ultrasound guidance over traditional techniques (nerve stimulation), particularly with respect to safety; ilioinguinal blocks may be an exception. Further studies are warranted [10, 11]. 

## 4. Neuraxial and Chronic Pain Procedures

Ultrasound has become a commonly used modality in the performance of chronic pain interventions and has begun to substitute for CT scans and fluoroscopy in many chronic pain procedures. It allows direct visualization of tissue structure while allowing real-time guidance of needle placement and medication administration. The following list summarizes some of current and potential applications of ultrasound in neuraxial and chronic pain procedures:

1. neuraxial blocks;
2. nerve root blocks (e.g., cervical and lumber);
3. stellate ganglion block;
4. lumber transforaminal injections for radicular pain;
5. facet joint block;
6. epidural blood patch;
7. intra-articular joint injections;
8. ultrasound guidance for peripheral nerve stimulator implantation;
9. interventional procedures for patients with chronic pelvic pain (e.g., pudendal neuralgia, piriformis syndrome, and “border nerve” syndrome).

Ultrasound can aid in neuraxial blocks in two ways: (1) ultrasound-assisted neuraxial technique and (2) real-time ultrasound-guided neuraxial technique. It helps in identification of landmarks and midline structures, estimating epidural space depth, and facilitating epidural catheter insertion [12]. Improvement in efficacy of epidural analgesia and technique difficulties are two other advantages of preprocedural ultrasound [13]. 

Karmakar et al. [14] in 14 out of 15 patients demonstrated successful use of real-time ultrasound-guided paramedian epidural access with in-plane needle insertion, without inadvertent dural punctures or complications. Real-time technique requires more expert personnel and a minimum of three hands, which may make it unpractical. 

Willschke et al. [15] evaluated ultrasound guidance for epidural catheter placement in children below six years, found that ultrasonography is helpful in reducing bone contacts, faster epidural placement, and offered direct visualization of neuraxial structures and the spread of local anesthetic inside the epidural space. Again, it needs highly skilled hands. 

Nerve root blocks under US guidance can be as effective as those placed using a fluoroscopy-guided method [16]. US facilitates identifying critical vessels at unexpected locations, thereby avoiding injury [17]. Transforaminal injection is a commonly used technique in management of spinal radicular pain. Ultrasound-guided transforaminal injection can be accurate and feasible in the preclinical setting, and it carries an advantage over traditional fluoroscopy or CT scan technique by avoiding radiation exposure and the ability to be performed as an outpatient procedure [18]. 

Ultrasound-guided facet joint block is another application that provides a minimal invasive procedure, with less time consumed, lower expenses, and fewer complications, in comparison with fluoroscopy-guided technique [19, 20].

Ultrasound-guided epidural blood patch allows confirmation of proper placement of injectate into the epidural space [21]. Clendenen et al. [22] presented a case series of six patients who were treated with 4-dimensional ultrasound-guided epidural blood patch for symptomatic postlaminectomy cerebrospinal fluid leak; all of them had relief of their headache. 

Ultrasound guidance of intra-articular joint injections (mainly the knee joint) improves needle placement and injection accuracy in comparison with palpation/anatomic landmark techniques, which improves patient-reported clinical outcomes and cost-effectiveness [23, 24]. Ultrasound-guided interventional procedures for patients with chronic pelvic pain (e.g., pudendal neuralgia, piriformis syndrome, and “border nerve” syndrome) were also reported [25].

## 5. Vascular Access

Advantages of ultrasound-guided central venous catheterization include identification of the vein, detection of variable anatomy and intravascular thrombi, and avoidance of inadvertent arterial puncture. It is safer and less time consuming than the traditional landmark technique [26, 27]. It is of particular benefit when used in patients with underlying coagulopathy or platelet dysfunction, by reducing the number of puncture attempts. Ultrasound can also be used for localization of central vein catheters and detection of postprocedural pneumothorax, as an alternative to chest radiography [28]. Ultrasound-guided vascular access has helped in various challenging patient positions: in sitting patients, patient with kyphosis and fixed chin-on-chest deformity [29], and in the prone position [30].

Ultrasound arterial cannulation helps in reducing the number of attempts, shortening the procedure time, and increasing the success rate, even in children [26]. A linear or hockey-stick probe can be used. However, it requires training to achieve a level of consistent proficiency. 

There is a marked reduction in complication rates after implementation of US-guided central venous cannulation approaches. Although some complications still happen, rates of 4.6% have been reported, comparing with 10.5% when using landmark technique, which represents an absolute risk reduction of 5.9% (95% CI 0.5–11.3%) [31]. Most of these complications occur due to inadequate operator's experience; “overshooting” the needle to exit the vein or failing to differentiate between vein and artery. French et al. [32] suggested a new 4-dimensional imaging (real-time 3-dimensional imaging) approach, using a matrix arrays transducer, for central venous cannulation, which shows promising results in preventing “overshooting” the needle and provides better visualization of anatomy. 

Peripheral vascular access in pediatrics can be very challenging especially in small, obese, or dehydrated children or in those with previously failed venipuncture. Studies showed that ultrasound-guided peripheral vascular access may improve the success rate of difficult vascular access when performed by well-trained physicians [33]. Recently, the High-frequency UltraSound in Kids studY (HUSKY) group, suggested that high-frequency (50 MHz) micro-ultrasound (HFMU) may allow better visualization for the sub-10 mm space. This could be a valuable tool for difficult vascular access in pediatric patients [34].

## 6. Airway Assessment

Airway ultrasound can visualize and assess the tongue, oropharynx, hypopharynx, epiglottis, larynx, vocal cords, cricothyroid membrane, cricoid cartilage, trachea, and cervical esophagus [4, 35, 36]. The posterior pharynx, posterior commissure, and posterior wall of the trachea cannot be visualized due to artifacts that are created by the intraluminal air column [35]. In comparison with computed tomography (CT scan), it has been found that ultrasound can reliably image all the structures that are visualized by CT scan and provides almost identical infrahyoid parameter measurements and minimal differences in suprahyoid anatomic parameter measurements, as the latter may be affected by the unintentional head extension [37].

Superficial structures can be scanned by linear high-frequency transducer, while deep structures are better visualized in sagittal and parasagittal views using the curved low-frequency transducer [4]. Current and potential applications of airway ultrasound are summarized as follows.

1. prediction of difficult airway;
2. confirmation of proper endotracheal tube placement and ventilation;
3. evaluation of airway pathologies that may affect the choice of airway management (e.g., subglottic hemangiomas and stenosis), or mandate urgent securing of airway (e.g., Epiglottitis);
4. prediction of obstructive sleep apnea;
5. prediction of size of endotracheal, endobronchial, and tracheostomy tubes;
6. airway related nerve blocks;
7. assessing and guidance for proper percutaneous dilatational tracheostomy (PDT);
8. prediction of successful extubation:
  - prediction of airway edema;
  - assessment of the diaphragm movement;
  - assessment of vocal cord movements.

Prediction of the difficult airway is a research area of great interest, with promising preliminary findings. Adhikari et al. [38] recently have reported that measurements of anterior soft neck tissue thickness at the level of the hyoid bone and thyrohyoid membrane can be used to predict difficult laryngoscopies, even though no significant correlation is found between sonographic measurements and clinical screening tests. An early study with a smaller sample size by Komatsu et al. [39] measured the distance from the skin to the anterior aspect of the airway at the level of the vocal cords, anterior to the thyroid cartilage, and failed to show a prediction of difficult laryngoscopy in obese patients. Thus, further studies are still needed in this area.

Confirmation of proper endotracheal tube placement can be done by two methods, direct and indirect [4, 40]. One direct method is the use of a real-time ultrasound probe placed transversely on the neck at the level of the suprasternal notch during intubation to observe whether the tube enters the trachea or esophagus. An indirect method is by observing bilateral lung sliding with ventilation as the probe is placed in the midaxillary line. Marciniak et al. [41] describe some characteristic ultrasonographic findings in the pediatric airway (e.g., shape changes of the glottis as the tracheal tube passes, enhanced posterior shadowing of the trachea, visualization of the vocal cords, and confirmation of bilateral lung movements) that could help during tracheal intubation. Recently, Fiadjoe et al. [42] reported an ultrasound-guided tracheal intubation in a 14-month-old baby, using a 15 MHz linear ultrasound probe at the level of the thyrohyoid membrane. They introduced (without laryngoscope) the tracheal tube containing a malleable stylet until it was visualized by ultrasound at the glottis level and then further adjusted the position and direction into the glottis until widening of the vocal cords was observed. 

Preliminary results of bedside ultrasonography show that it is a safe and effective tool to diagnose acute epiglottitis. In two recent studies, Hung et al. [43] visualized the “P sign" in a longitudinal view through the thyrohyoid membrane. Ko et al. [44] found a significant difference in the anteroposterior diameter of the epiglottis in acute epiglottitis patients. These findings can facilitate early and proper airway management. 

Diagnosis and prediction of obstructive sleep apnea is a challenge, as many patients come for surgery undiagnosed. Lahav et al. [45] found that tongue base width, measured by ultrasound, may influence the severity of obstructive sleep apnea, including the patients' sensation of choking during night. Another important correlation, found by Liu et al. [46], is the lateral parapharyngeal wall thickness. Further studies are still needed in this area.

Ultrasonography has been used successfully to guide the choice of the appropriate size of endotracheal tube [4, 47], tracheostomy tube [4, 36], and even double-lumen tube [48, 49]. Ultrasound is successfully improving the performance of airway related nerve blocks [36], including superior laryngeal nerve, deep cervical plexus, alveolar nerve, and superficial trigeminal nerve. Kaur et al. [50] recently published their preliminary results in ultrasound-guided superior laryngeal nerve block for upper airway anesthesia, using a hockey stick-shaped 8 to 15 MHz transducer and concluded that it is a feasible approach.

Percutaneous dilatational tracheostomy (PDT) is a frequent procedure in intensive care units [36], with possible potential complications, like hemorrhage from local vessels and tracheal stenosis, due to a higher placement of the tracheostomy. Advantages of ultrasound in this setting include [4, 36, 51] identification of possible vessels in the field and localization of the midline and the tracheal rings for optimal intercartilaginous space selection, to avoid any possible laryngotracheal stenosis. The distance from the skin to the surface of the trachea can also be measured in order to estimate the required length of the puncture cannula. Another approach for using real-time ultrasonic guidance with visualization of the needle has been reported [52] and appears to be feasible, accurate, and safe. Selecting the good candidate seems to be the main advantage of ultrasound PDT. 

Prediction of successful extubation is another challenge, especially in long-term intubated patients and in those who have a high risk of airway edema and vocal cord injuries (e.g., after thyroid surgery). A pilot study by Ding et al. [53] reported a useful method for predicting postextubation stridor. They found that the air-column width during cuff deflation at the level of the cricothyroid membrane is a potential predictor of postextubation stridor that reflects laryngeal edema. Jiang et al. [54] found that the cranio-caudal displacement of the liver and spleen with a cut-off value of 1.1 cm during spontaneous breathing trials, measured by ultrasonography, is a good predictor for extubation outcome. 

**Figure 2:**
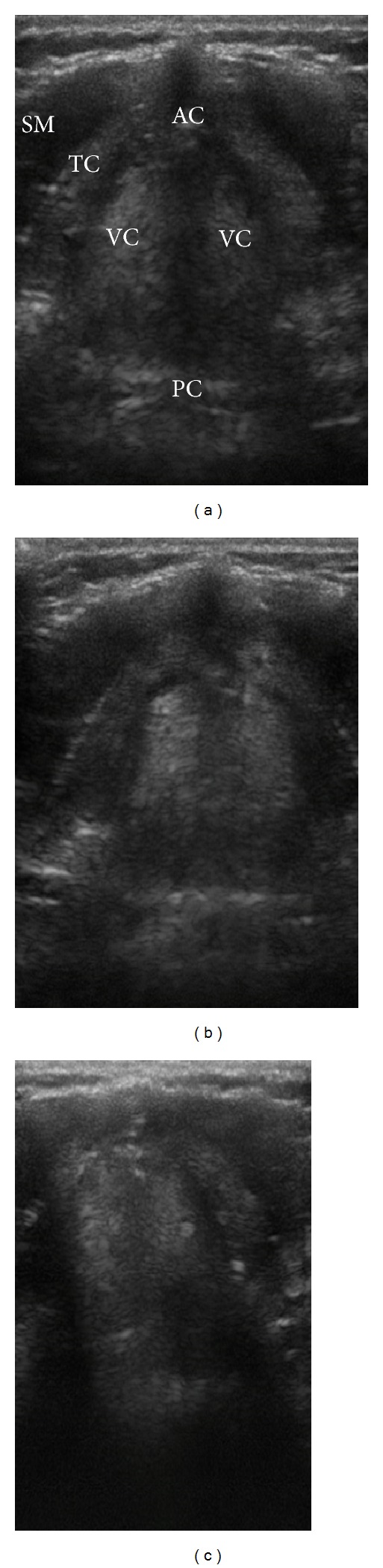
Vocal cords assessment: SM: strap muscles; TC: thyroid cartilage; AC: anterior commissure; PC: posterior commissure; and VC: vocal cords. (a) Vocal cords are abducted on inspiration, (b) adducted partially during expiration, (c) and are tightly closed when asking the patient to say “Eeeee.” Linear transducer was placed transversely on the midline of the cricothyroid membrane.

Laryngeal ultrasound (Figure 2) to assess vocal fold paralysis in children has been suggested as a useful adjunct to endoscopy in diagnosis of vocal cord palsy [55]. Shaath et al. [56] assessed the accuracy of US in detection the vocal cord mobility in children after cardiac surgery in comparison with standard fiber-optic laryngoscopy and reported a sensitivity of 100% and specificity of 80% in 10 patients with persistent significant upper airway obstruction. A recent case report [57] shows a successful detection of recurrent laryngeal nerve palsy in the immediate postoperative period after thyroid surgery. Although endoscopy is still considered the gold standard for diagnosis of vocal cord palsy, the noninvasive nature and portability make ultrasound a good screening tool pre- and postthyroidectomy. 

## 7. Lung Ultrasound

In a number of emergency situations, hypoxia will require urgent and appropriate diagnosis for its management. Pneumothorax, pulmonary edema, pulmonary embolism, and ARDS are situations where ultrasound can be an important tool for diagnosis (as shown in the following list). Lichtenstein et al. [58] introduced a quick and accurate ultrasound protocol (BLUE protocol) for a rapid diagnosis and differentiating the cause of acute respiratory failure in critical care settings. We believe that a similar protocol could possibly be applied to our anesthetized patients. Lung ultrasound has a higher diagnostic yield than chest X-ray for most of the aforementioned conditions [59]; it is easier to carry out and less time consuming. However, it has some limitations when used in patients with subcutaneous emphysema, pleural calcifications, and in the obese [5]. Current and potential applications of lung ultrasound are as follows.

1. diagnosis of pneumothorax;
2. diagnosis of interstitial syndrome;
3. diagnosis and differentiation of underlying cause of Pleural effusion, and selecting the optimal puncture site for pleurocentesis;
4. diagnosis of pulmonary consolidation and pneumonia;
5. diagnosis of atelectasis
6. diagnosis of pulmonary edema and differentiate it from acute respiratory distress syndrome (ARDS);
7. diagnosis of pulmonary embolism;
8. monitoring of lung disease (severity, progress, and response to therapy);
9. optimizing mechanical ventilation.

A high frequency (7.5 to 10 MHz) transducer is an appropriate choice for detecting pleural line abnormalities, while lower frequency (3.5 MHz) convex and microconvex transducers can be used to diagnose pleural effusions and lung parenchymal abnormalities. [5]. B- and M-mode may be used during lung ultrasound scanning and the produced sonographic images are a virtual interplay of two elements: air and fluid. Lung ultrasound interprets mainly the presence or absence of various artifacts since air is an acoustic barrier.

### 7.1. Normal Lung Aeration Patterns Reflect Specific Sonographic Signs [5] (Figures 3(a) and 3(b))

- “Lung sliding” signs are sliding of visceral and parietal layers of pleura with respiration.
- Seashore sign is a complex picture of parallel lines signifying the static thoracic wall and sandy “granulous” pattern, which reflect the normal pulmonary parenchyma.
- A-lines are a basic artifact of normally aerated lung.

### 7.2. Pathological Lung Signs and Patterns Include the following [5]

- B-lines represent discrete laser-like vertical hyperechoic lines that arise from the pleural line and extend to the bottom of the screen. These lines are consistent with interlobular pulmonary edema and can be found in both ARDS and cardiogenic pulmonary edema.
- Dynamic and static air bronchograms which consist of hyperechoic punctiform elements within the lung parenchyma can be used to diagnose consolidation and atelectasis, respectively.
- Lung pulse is an early and dynamic diagnostic sign of complete atelectasis, in which US perceives the vibrations of heart activity, along with the absence of lung sliding [60].

*The International Liaison Committee on Lung Ultrasound (ILC-LUS) *has recommended the following signs for the detection of various lung abnormalities [59].

**Figure 3:**
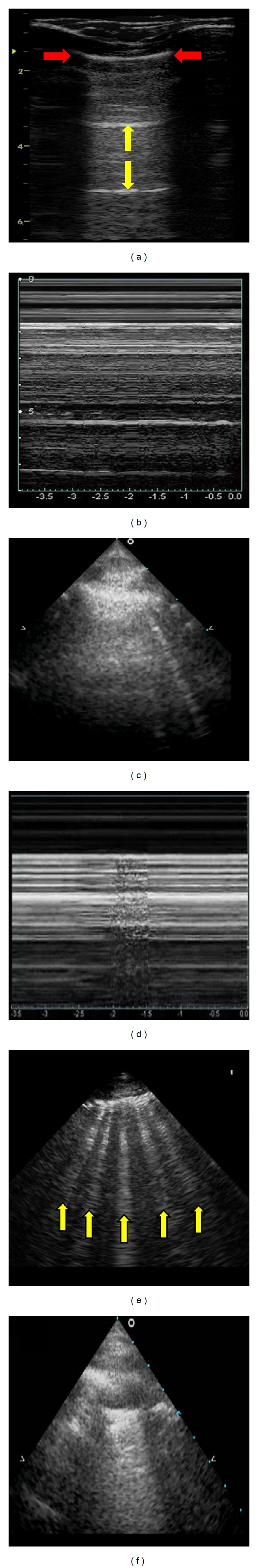
Normal lung ultrasound: (a) 2D “red arrows” point to the pleura, where the normal “sliding sign” should be seen, while the “yellow arrows” represent the A-lines that are normal reverberation from the pleura. (b) M-mode shows the “seashore sign.” Pneumothorax: (c) 2D; absence of lung sliding, (d) M-mode; “stratosphere sign” or “barcode sign,” lung point may also be seen during inspiration and represents the border between pneumothorax and normal pleura. Cardiac pulmonary edema: (e) homogeneous distribution of B-lines (yellow arrows), normal sliding, and no spared areas. *Acute *respiratory distress syndrome (ARDS): (f) “patchy” distribution of B-lines, reduced/abolished sliding, spared areas, and peripheral consolidations.

*(i) Pneumothorax (Figures 3(c) and 3(d)).* Absence of lung sliding, presence of lung point(s), absence of B-lines, and absence of lung pulse. Lung ultrasound rules out the diagnosis of pneumothorax more accurately than a supine anterior chest X-ray (evidence level A). 

*(ii) Interstitial Syndrome (Figures 3(e) and 3(f)).* Presence of a B-profile consisting of more than 3 B-lines on a longitudinal scanning plane. Interstitial syndrome includes pulmonary edema, interstitial lung disorders and ARDS (evidence level B). [59, 61]. 

*(iii) Lung Consolidation.* Sonographic signs are a subpleural echo-poor region or one with tissue-like echotexture. Lung ultrasound can differentiate between consolidation of pulmonary embolism, pneumonia, and atelectasis (evidence level A). 

*(iv) Pleural Effusion.* A hypoechoic or anechoic space between sonoanatomical boundaries (i.e., chest wall, the diaphragm and subdiaphragmatic organs). Lung ultrasound is more accurate than chest X-ray (evidence level A). 

*(v) Monitoring Interstitial Syndrome.* The number of B-lines is directly proportional to the severity of pulmonary congestion. This could be used as a monitoring parameter of severity and response to therapy (evidence level A). Pulmonary edema can be diagnosed, quantified, and monitored by detection of B-lines [62]. 

**Figure 4:**
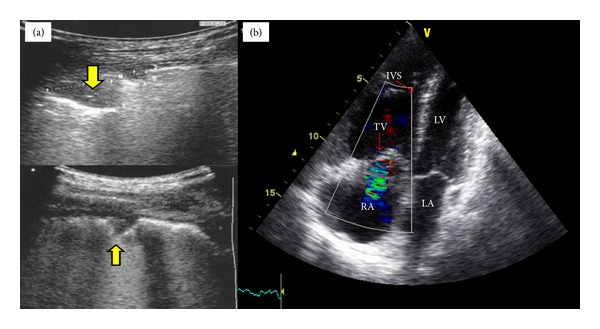
Pulmonary embolism: ((a) lung ultrasound) peripheral, triangular, and pleural based hypoechoic lesions (yellow arrows); ((b) transthoracic echo, apical view) it shows right ventricular (RV) dilation, RV hypokinesia, septal flattening, and tricuspid regurgitation. IVS: interventricular septum; TV: tricuspid valve; LV: left ventricle; RA: right atrium; LA: left atrium.

Pulmonary embolism (PE) (Figure 4), “mainly peripheral” can be diagnosed sonographically by the recognition of a peripheral, triangular, and pleural based hypoechoic lesion [5]. Mathis et al. [63], in a multicenter study that involves 352 patients, defined diagnostic criteria as (1) PE confirmed: two or more typical triangular or rounded pleural-based lesions; (2) PE probable: one typical lesion with pleural effusion; (3) PE possible: small (<5 mm) subpleural lesions or a single pleural effusion only. The sensitivity was 74%, specificity 95%, positive predictive value 95%, negative predictive value 75%, and accuracy 84%. 

Laursen et al. [64] have studied the utility of lung ultrasound in near-drowning victims. Lung ultrasound showed multiple B-lines on the anterior and lateral surfaces of both lungs, consistent with pulmonary edema. These findings may encourage anesthesiologists to consider lung ultrasound for diagnosing aspiration pneumonia during anesthesia. 

During positive pressure ventilation (PPV) a quantitative assessment of B-lines may aid in guiding diuretic or optimizing ventilator settings, particularly in conditions such as pulmonary edema or increased lung water content [65]. In general, PPV supports the function of an impaired left ventricular (LV) by reducing the transmural pressure across the LV free wall (LV afterload is reduced). In contrast, PPV is usually a functional burden on an already impaired right ventricle (RV) function, due to the reduction of preload and increase in afterload, respectively. Ultrasound can help in optimizing PPV to achieve the maximum benefit in oxygenation while avoiding its side effects on cardiac function. Ultrasound-guided optimization of positive end-expiratory pressure (PEEP) [66] is a clear example of this beneficial tool. PEEP can be titrated up and followed by quantifying the number of B-lines while watching RV filling and assuring that this PEEP is not causing any decrease in RV filling. Thus chest ultrasound (lung and cardiac ultrasound) evaluation can guide both ventilator and circulatory support. The optimization of heart-lung interaction can enhance the therapeutic effect of mechanical ventilation and facilitates the weaning process as well [67].

## 8. Ultrasound Neuromonitoring

Elevated intracranial pressure (ICP) requires special precautions by the anesthesiologist, such as avoiding particular medications, ventilation settings, and neuraxial anesthesia. Ultrasound is useful in assessing elevated ICP and cerebral perfusion. Current and potential applications of neuroultrasound are as follows:

1. optic nerve sheath diameter (ONSD) measurement;
2. transcranial Doppler ultrasound (TDU);
3. pupillary light reflex (PLR).

**Figure 5:**
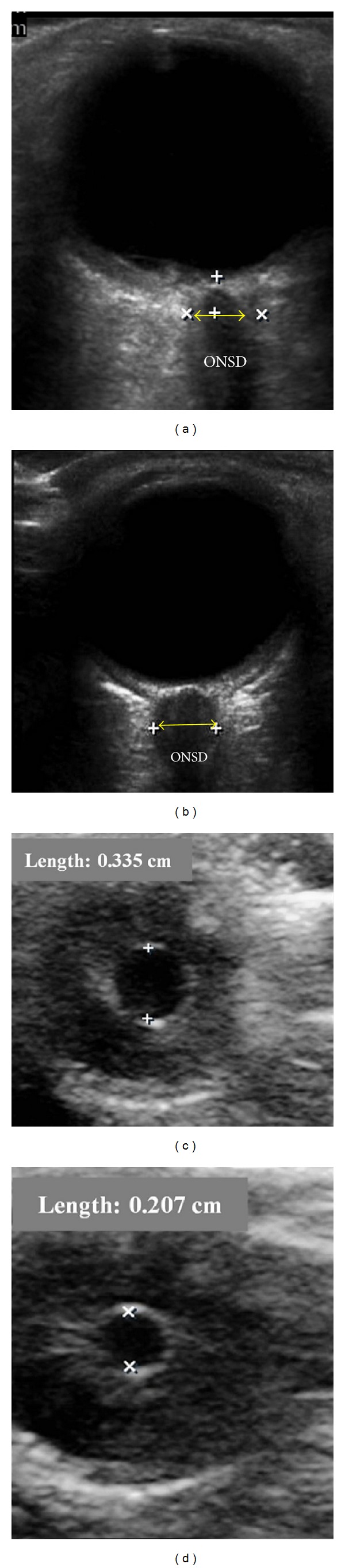
Optic nerve sheath: (a) normal diameter and (b) large diameter that represents increase intracranial pressure. ONSD: optic nerve sheath diameter. It is usually measured 3 mm behind the retina. Ultrasound pupillary light reflex: (c) diameter of the pupil before shining light to the contralateral eye and (d) the pupil constricted after shining the light.

Measurement of optic nerve sheath diameter (ONSD) (Figures 5(a) and 5(b)) has been found to reflect intracranial pressure, as an increase in ICP will be transmitted through the subarachnoid space that surrounds the optic nerve within its sheath and has been proposed as noninvasive and reliable means of assessing ICP in neurocritically ill patients [68]. 

In a recent systematic review and meta-analysis [69], ONSD measurements exhibited a pooled sensitivity of 0.90 (95% CI 0.80–0.95) and specificity 0.85 (95% CI 0.73–0.93) in detecting elevated ICP, while the area under the summary receiver-operating characteristic (SROC) curve was 0.94 (95% CI 0.91–0.96).

Soldatos et al. [70] found that 5.7 mm is a cut-off value for elevated ICP with sensitivity of 74.1% and specificity of 100%. The main drawbacks to this technique are operator dependence and measurement of relatively small structures, while technical improvements may be necessary. 

Dubost et al. [71] measured ONSD in transverse and sagittal plane in patients with preeclampsia and compared the findings with those obtained in healthy pregnant women. They found that median ONSD values were significantly greater in preeclamptic patients at delivery (5.4 mm (95% CI 5.2, 5.7) versus 4.5 mm (95% CI 4.3, 4.8), *P* < 0.0001). In about 20% of preeclamptic patients, ONSD reflected compatible values with intracranial pressure above 20 mmHg. Further studies are required to improve the technique of this useful ultrasound methodology. 

It has been thought [72] that intracranial hypotension, as in a setting of dural leak, might be associated with decreased ONSD, as the optic nerve is surrounded by cerebrospinal fluid and dura mater, which form the optic nerve sheath. Dubost et al. [73], in a preliminary report of 10 patients with lumbar epidural blood patch (EBP) for postdural puncture headache, indeed found that successful EBP was followed by ONSD enlargement.

Ultrasound assessment of the pupillary light reflex (PLR) was initially developed for the U.S. Space Program (NASA) and is not standardized for clinical use. However, the method can be used even when visual access to the pupil is impossible, and interpreting its results is straightforward [74]. Consensual pupillary light reflex is elicited with contralateral transillumination through the eyelids with both eyes closed (Figures 5(c) and 5(d)). The pupillary light reflex ultrasound test can be conducted with a linear array probe at the highest available frequency (e.g., 12–15 MHz), using the coronal primary view, while M-mode measurements are used to measure the constriction velocity of the PLR [75]. This method might be used as pupillometry as well. 

**Figure 6:**
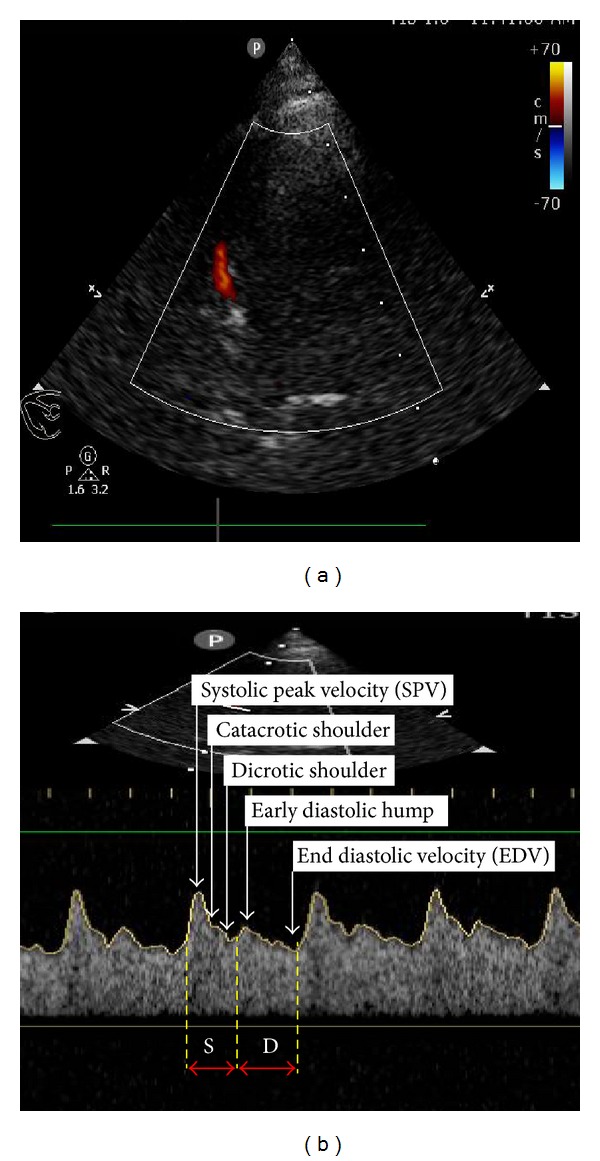
Transcranial color coded duplex (TCCD): (a) middle cerebral artery (MCA) color Doppler and (b) MCA pulsed wave Doppler. S: systole, D: diastole.

Transcranial color coded duplex (TCCD) (Figure 6) is an accurate, real-time, noninvasive (permits bedside examination), and inexpensive tool used for the study of the intracranial circulation and the diagnosis of nonthrombosed aneurysms, largely due to its ability to reveal flow phenomena [76]. The main limitation of TCCD is the few available ultrasonic windows, which can limit the area of insonation of the cerebral arteries including their proximal branching and lower spatial resolution and can obstruct transtemporal insonation [77]. TCCD has advantages over transcranial Doppler (TCD) by showing the images of the intracranial anatomy and arteries throughout duplex B-mode, while still having the capacity to measure velocities using Doppler. In other words, different from TCD technology, TCCD shoots multiple ultrasound beams to expose a larger brain area at dual emitting frequencies, one for gray scale imaging and one for Doppler imaging. Thus this tool can illustrate arterial position on color flow imaging as well as on B-mode ultrasonography [78]. TCD and TCCD measured velocities are comparable using zero angle correction, resulting in more accurate measurement of flow velocities and allowing for superior precision in order to define intracranial arterial narrowing. TCCD can be used for monitoring of cerebral blood flow alterations which follow traumatic brain injury and in patients with sickle cell anemia. It also can be used in the detection of patent foramen ovale and in the diagnosis of cerebral circulatory arrest which is a component of brain death [79].

## 9. Gastric Ultrasound

A full stomach may lead to aspiration pneumonia and subsequent morbidities. Anesthesiologists may encounter patients with unknown prandial status, and even fasting “sufficient” time cannot guarantee an empty stomach in many cases (e.g., in the elderly or in patients with gastroparesis). Ultrasound can help in this setting, and the perioperative evaluation of bowel motility is also feasible by means of sonography. Current and potential applications of Gastric ultrasound are as follows:

1. assessment of gastric content and diagnosis of full stomach;
2. confirmation of gastric tube placement.

Bouvet et al. [80], measured the antral cross-sectional area (CSA) in 180 patients after intubation and analyzed the relationship between antral CSA and the volume of gastric contents. The cut-off value of antral CSA of 340 mm^2^ for the diagnosis of “at risk” stomach was associated with a sensitivity of 91% and a specificity of 71%. The area under the receiver operating characteristic (ROC) curve for the diagnosis of “at-risk” stomach was 90%. (“At risk” stomach was defined as the presence of solid particles and/or gastric fluid volume more than 0.8 mL/kg.) These findings show that antral CSA volume assessment can be important in minimizing the risk of pulmonary aspiration of gastric contents. Perlas et al. [81] performed gastric sonography in 86 patients before anesthetic induction, and patients were classified using a 3-point grading system; grade 0 (empty antrum); grade 1 (minimal fluid volume detected only in the right lateral decubitus position (16 +/− 36 mL, within normal ranges expected for fasted patients); and grade 2 (antrum clearly distended with fluid visible in both supine and lateral positions (180 +/− 83 mL, beyond previously reported “safe” limits). One patient with a grade 2 antrum had an episode of a significant regurgitation of gastric contents on emergence from anesthesia. They concluded that this grading system could be a promising “biomarker” to assess perioperative aspiration risk. Perlas et al. [82], in another work, validated a mathematical model for quantitative US assessment of gastric volume. Arzola et al. [83] found that anesthesiologists will achieve a 95% success rate in bedside qualitative ultrasound assessment after performing approximately 33 examinations, with appropriate training and supervision. 

Confirmation of a gastric tube placement is also possible using ultrasound [84], which might replace the conventional radiography method unless sonography is inconclusive.

## 10. Focused Transthoracic Echo (TTE)

**Figure 7:**
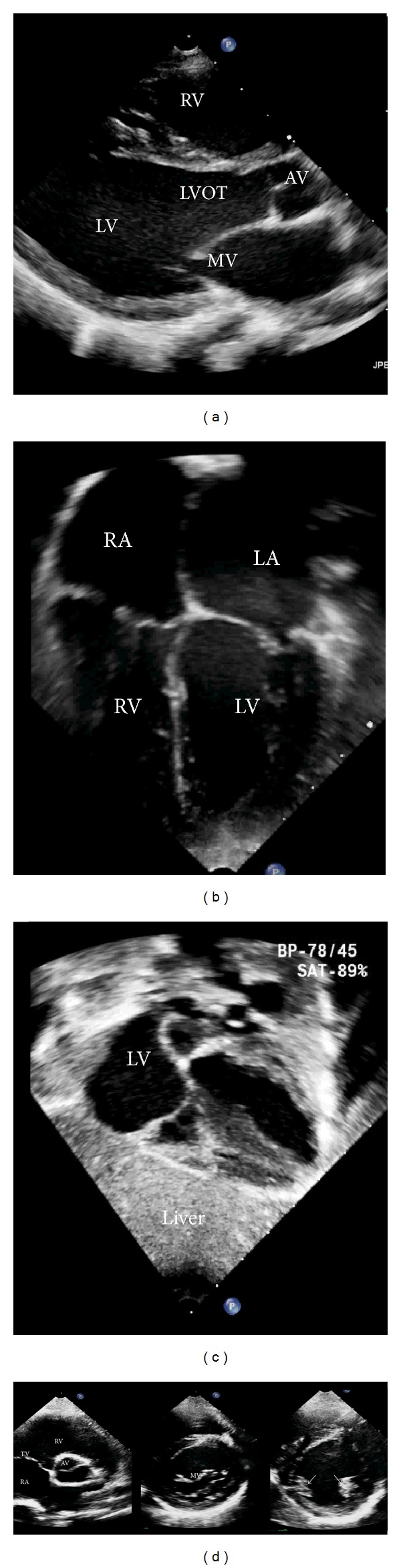
Basic transthoracic echo views: (a) left parasternal long axis, (b) apical, (c) subcostal, and (d) left parasternal short axis; aortic valve “Mercedes sign,” mitral valve “fish mouth sign,” and papillary muscles (two arrows), respectively, from left to right. RV: right ventricle, LV: left ventricle, LVOT: left ventricular outlet, RA: right atrium, LA: left atrium, AV: aortic valve, MV: mitral valve, and TV: tricuspid valve.

Focus assessed transthoracic echo (FATE) was introduced by Jensen et al. [85] for cardiopulmonary monitoring in the intensive care unit. This approach basically involves four standardized acoustic views for cardiopulmonary screening and monitoring (Figure 7). Recent studies show a great impact of FATE in preoperative assessment [86, 87] when it is performed by anesthesiologists. Dennis and Stenson [88], recently presented a case that shows how anesthesiologists, using basic TTE skills, can diagnose and save a patient with postpartum hemorrhage by using a rapid obstetric screening echocardiography approach [89]. Learning the basic skills to perform focused transthoracic echo allows assessing the global function of the heart and diagnosing certain pathologies (e.g., pulmonary embolism). Cowie [90] presented their 3-year experience of focused cardiovascular ultrasound in the perioperative period, which shows that focused cardiovascular ultrasound performed by anesthesiologists in the perioperative period accurately detects major cardiac pathology and significantly alters perioperative management. Neelankavil et al. [91] suggested a simulation method to train anesthesiologists in basic transthoracic echocardiography skills. Tanzola et al. [92] have suggested that implementation of a focused bedside TTE curriculum within anesthesia residency training is feasible, quantifiable, and effective for increasing anesthesia residents' TTE knowledge.

Recent studies show that preoperative excess testing and consultation are common, adding to the cost of care without noticeably improving patient outcome. These findings must encourage anesthesiologists to play an effective role in the preoperative assessment field by implementing clinically innovative approaches and developing training curricula as well as performing research [93]. Therefore, we strongly encourage the use of a focused protocol for perioperative assessment and incorporation of a training curriculum in residency training.

## 11. Technological Advances 

New technologies have greatly improved the image quality, diagnostic abilities, and size of the US machine. These include advances in transducers, scanning schemes, three- and four-dimensional visualization, contrast agents (microbubbles), strain imaging, and others [94]. 

The matrix array is a new transducer with improved resolution; it has a lens that is placed in front of the piezoelectric element to allow a mechanical focusing in the *Y*- and *Z*-planes. Four-dimensional ultrasound provides real-time 3D images (the 4th “D” is time) and currently is used for fetal imaging, where it provides remarkable images. It may have potential applications in our field. Endobronchial and endoscopic ultrasounds are two other new modalities with great and potential implications [95]. 

Recently, the use of three-dimensional high resolution ultrasound was reported [96] for nerve blockade, providing better visualization of local anesthetic spread and catheter tip location. Small and portable ultrasound systems have become increasingly available, even a mobile ultrasound-guided peripheral nerve block has been developed [97]. The SonixGPS needle guidance system (Ultrasonix, Richmond, BC, Canada) is a GPS technology with a new needle tracking system, using sensors in both the needle and transducer to obtain a real-time image of needle shaft and tip position related to the ultrasound beam that is based on the needle trajectory. This can be used for vascular access and nerve blocks. Recently, it has been used for real-time thoracic paravertebral block [98] and spinal anesthesia [99] in pilot studies.

## 12. Conclusion 

Ultrasound is a unique tool which provides the anesthesiologist with diagnostic and monitoring capabilities enabling optimization of perioperative management. Indeed, ultrasound has an important role in problem-based management of various anesthesiology emergencies such as hypoxia, hypotension, dyspnea, and cardiopulmonary arrest. Finally, procedural ultrasound applications in the field of anesthesiology are numerous and improve the quality of care. 

We believe that ultrasound can be the third eye of the anesthesiologist that helps in the performance of previously blind procedures and allows discovery of many hidden spaces to uncover their mysteries. Anesthesiologists, in the near future, may need to carry a portable ultrasound around their neck instead of a stethoscope.
